# Vitamin B6 Levels and Impaired Folate Status but Not Vitamin B12 Associated with Low Birth Weight: Results from the MAASTHI Birth Cohort in South India

**DOI:** 10.3390/nu15071793

**Published:** 2023-04-06

**Authors:** R. Deepa, Siddhartha Mandal, Onno C. P. Van Schayck, Giridhara R. Babu

**Affiliations:** 1 Indian Institute of Public Health-Bangalore, Public Health Foundation of India (PHFI), Bengaluru 560023, India; 2 Centre for Chronic Disease Control, Public Health Foundation of India (PHFI), Gurgaon 122002, India; 3 Care and Public Health Research Institute, Maastricht University, P.O. Box 616 Maastricht, The Netherlands; 4 DBT Wellcome Trust India Alliance, Hyderabad 500034, India

**Keywords:** B12 deficiency, vitamin B6, folic acid, pregnancy, low birth weight, preterm delivery

## Abstract

Vitamins B12 and B6 and folate are known to have implications for pregnancy outcomes. We aimed to describe B6, B12, and folate status in pregnancy and investigate their associations with low birth weight and preterm delivery in mothers recruited from public hospitals in urban Bengaluru. Pregnant women between 18 and 45 years were included in the MAASTHI prospective cohort study. Each participant’s age, socioeconomic status, and anthropometry were recorded during baseline and followed up after delivery. Blood samples were collected between the 24th and 32nd weeks of gestation and stored at −80° for analysis. B6, B12, folate, homocysteine, and methylmalonic acid (MMA) levels were analyzed in the stored samples. We found low plasma vitamin B12, folate, and B6 levels in 48.5%, 42.0%, and 10.4% of the women (*n* = 230), respectively. Elevated MMA and homocysteine were observed among 73.6% and 6.1% of the women, respectively. We found B6 levels were significantly associated with birth weight (β(SE) −0.002(0.0), *p* = 0.001) after adjusting for age, parity, adiposity, gestational diabetes, and socioeconomic status of the mother. Those with impaired folate deficiency were twice at risk (AOR 1.95 (1.29, 3.07), *p* = 0.002) of low birth weight. Vitamin B6 levels and impaired folate status were associated with low birth weight in the MAASTHI birth cohort.

## 1. Introduction

In 2015, 20 million children (14.6%) were born with low birth weight, and nearly half were born in Southern Asia [1]. Regional trends in low- and middle-income countries show the highest annual LBW rates in Asian sites at 20.2%, followed by Central American sites at 15.6% [2]. The prevalence of low birth weight (LBW) is India’s major public health issue, with four out of ten babies with LBW in the developing world being born in India [1]. A meta-analysis of studies published from 2004 to 2014 reported a 31% adjusted pooled estimate for the prevalence of low birth weight in India [3]. However, it is reassuring to see significant declines in low birth weight in the past decade, from 25.2% in the 1992 National Family Health Survey (NFHS) to 17.5% in 2015–16 [4]. Babies with LBW are prone to short- and long-term health problems. LBW is a major cause of neonatal mortality and morbidity. In addition, infants are also at high risk for metabolic and cardiovascular diseases in later life.

Mothers’ nutritional status is the most important determinant of newborn children’s birth weight. Women with low hemoglobin values, low body mass index, and height less than 150 cm are more likely to give birth to low-birth-weight babies. Pregnancy-induced physiologic changes and the growing fetus increase the micronutrient demand. Studies have shown that micronutrient deficiency is strongly associated with low birth weight; mothers not receiving supplementation are at higher risk of delivering a low-birth-weight child [5].

Water-soluble B vitamins folate, vitamin B6, and vitamin B12 play key roles as enzyme cofactors or substrates in one-carbon metabolism (OCM), a process whereby folate transfers one-carbon groups in a series of biological processes. These processes encompass DNA synthesis, methylation, and metabolism of homocysteine. Therefore, disturbance of OCM due to altered nutrient status is associated with aberrant DNA methylation patterns and several adverse outcomes such as birth defects, cancer, metabolic disorders, and cardiovascular disease [6]. Interestingly, the Pune Maternal Nutrition Study showed no association between energy, protein intake, and LBW. At the same time, there was a strong association between folate levels and foods rich in micronutrients [7]. However, there is still a limited understanding of the specific nutrients important for fetal growth and development.

Nationwide data on the prevalence of multiple micronutrient deficiencies during pregnancy are not readily available in India. Vitamin B12 deficiency is highly prevalent among Indians, as many vegetarians lack access to foods rich in this vitamin. The Comprehensive National Nutrition Survey (2016–18) showed that 26.8% and 34.1% of female adolescents had vitamin B12 and folate deficiencies [8]. Some studies have reported a high prevalence of vitamin B12 deficiency during pregnancy (40–70%) [9,10]. Vitamin B12 deficiency in pregnancy has been associated with both lower birth weight (birth weight <2500 g) and preterm birth (length of gestation <37 weeks). Certain folate species levels have been associated with a shorter duration of pregnancy [11]. Reports on the prevalence of B6 deficiency are scarce in India. The risk of preterm birth was 50% higher among Chinese women who were vitamin B6 deficient [12].

Investigating the role of nutrients influencing OCM in India is important. Moreover, very little is known about the relationship between micronutrient status and birth outcomes in women attending public health facilities. We explored vitamin B6, B12, and folate levels in pregnancy and investigated their association with low birth weight and preterm delivery.

## 2. Materials and Methods

### 2.1. Design

The study is nested within an ongoing study, the ‘Maternal Antecedents of Adiposity and Studying the Transgenerational Role of Hyperglycaemia and Insulin’ (MAASTHI) cohort, a prospective pregnancy cohort in public hospitals of Bengaluru. Women are recruited during pregnancy and followed up from delivery until the child is four years old. MAASTHI was set up to understand the effect of maternal nutritional and psychosocial environment on the child’s noncommunicable disease risk factors. Around 2962 pregnant women were recruited from public hospitals from 14 to 32 weeks from 2016 to 2019; after obtaining consent, a baseline questionnaire was administered. Blood samples were collected between 24 and 32 weeks in a subsample of randomly selected pregnant women (*n* = 230). The sample size for studying the association between vitamins B12 and B6, folate, and low birth weight was estimated based on a prevalence of 18% low birth weight [13], with a 95% confidence interval using 5% precision. The sample size obtained is 227. A total of 230 samples were randomly chosen from the MAASTHI cohort to perform the micronutrient assessment in the stored samples.

### 2.2. Data Collection

Sociodemographic details, obstetric history, and family history of noncommunicable diseases (NCDs) were collected from pregnant women during the baseline visit between 14 and 32 weeks by administering a questionnaire. Data were collected only once during pregnancy. Socioeconomic status was assessed based on the Kuppuswamy scale of classification using education, occupation, and income, into Upper, Upper middle, Lower middle, Upper lower, and Lower. For analysis purposes, only two main categories of lower (Upper, lower, and Lower) and middle (Upper middle, Lower middle) were created.

### 2.3. Anthropometric Measurements

In pregnant women, height in centimeters was measured to the nearest 0.1 cm using a portable stadiometer (SECA 213). Weight was measured to the nearest 0.1 kg using digital weighing. BMI was classified as Normal (18.5–22.9), Underweight (<18.5), Overweight (23–24.9), and Obese (≥25). At birth, infants’ age, gender, weight, length, and circumferences were measured. Weight was recorded to the nearest 10 g using the SECA 381 baby weighing scale. Infants’ birth weight of less than 2.5 kg was categorized as low birth weight.

### 2.4. Biochemical Analyses

Between 24 and 32 weeks of gestational age, an oral glucose tolerance test was conducted among pregnant women, and hemoglobin was estimated on an automated analyzer. Based on the WHO criteria, a hemoglobin concentration of 110 g/L or higher is classified as nonanemic, and less than 109 g/L is classified as anemic [14]. The oral glucose tolerance test (OGTT) was conducted to diagnose gestational diabetes mellitus (GDM) after overnight fasting. A venous blood sample was drawn to estimate fasting blood sugar (FBS). The pregnant woman was then given a 75 g oral glucose solution, and a second venous blood sample was collected 2 h later for estimating postprandial blood sugar (PPBS). GDM was diagnosed if FBS ≥92 mg/dL and/or PPBS ≥153 mg/dL [15]. A 6 mL blood sample was collected in a plain vacutainer from each participant for serum preparation by venipuncture procedure by a trained phlebotomist. The vacutainer was wrapped with aluminum foil to protect it from sunlight and kept for 45 min to coagulate. These were then centrifuged for 10 min at 1500 rpm/min. Clear supernatant serum was transferred into black-color cryovials. Serum samples were stored at −80 degrees in a biorepository. A total of 230 samples were randomly analyzed for vitamin B12, serum folate, methylmalonic acid, and homocysteine in the stored samples. These samples were shipped to the laboratories in dry ice for conducting assays. Vitamin B12 and folic acid concentrations were determined using the serum/chemiluminescent microparticle immunoassay (CMIA) assay method in a fully automated analyzer (Abbott ARCHITECT i2000/i2000SR. Internal and external quality check was performed; the External Quality Assurance Scheme (EQAS) coefficient of variation (%) for B12 was 12.7, and for folate, it was 13.6. Quality-control samples on low, middle, and high ranges of vitamin B12 were analyzed along with the samples.

The vitamin B12 cut-offs are as follows: >221 pmol/L is vitamin “B12 adequacy”, between 148 and 221 pmol/L is “low B12”, and lower than 148 pmol/L is “B12 deficiency” [16].

Folate was considered as low if <10 nmol/L (4 ng/mL) [17].

Homocysteine was analyzed using the chemiluminescence method, and vitamin B6 and methylmalonic acid were analyzed through serum/ultra-high-performance liquid chromatography–tandem mass spectrometry. Homocysteine was elevated if it was >10 μmol/L, and MMA above 0.26 μmol/L was considered high. MMA (>0.26 μmol/L) was used to reflect depleted vitamin B12 status. Vitamin B6 >30 nmol/L was considered adequate, 20–30 nmol/L suboptimal, and <20 nmol/L was B6 deficiency.

Combined vitamin B12 (cB12), a composite indicator of vitamin B12 status, modified for three biomarkers (i.e., vitamin B12, MMA, tHcy), was calculated using the formulas developed by Fedosov et al. [18].

### 2.5. Statistical Analysis

The normality of variables was tested using a histogram and the Shapiro–Wilk test. Quantitative variables are described as mean ± standard deviation (SD). Nutritional markers are also reported as median and IQR, and qualitative variables are reported as percentages. Respondents were divided into two groups: LBW children and normal-weight children.

We performed univariable linear regression and multivariable linear regression analysis models adjusted for confounders in the association between LBW, preterm delivery, and maternal characteristics.

We created a composite variable termed ‘Impaired folate status’ (high Hcys and low folate) to identify women with confirmed folate deficiency. Associations between the composite exposure variables and the prevalence of low birth weight were analyzed using a causal model with inverse probability treatment weighting using propensity scores. Propensity scores were calculated using predicted probabilities from logistic regression models of composite exposure variables against the confounding variables.

Based on a priori knowledge, maternal age, parity, BMI and socioeconomic status, maternal adiposity and gestational diabetes mellitus, and sex of the child were included as confounders. Maternal age ≤20 years, nulliparity, and low socioeconomic status are known causative factors for the high incidence of LBW. Male gender has a protective effect against LBW, and so does maternal adiposity and gestational diabetes mellitus.

All analyses were performed using SPSS software version 20 (SPSS, Chicago, IL, USA) and R software version 4.0.

## 3. Results

The mean age of the participants in the study was 24.2 years. The baseline assessment was conducted at around 22.5 weeks, and the blood sample was drawn between 24 and 32 weeks. Most of the women had attended pre-university college (41.9%). Around one-third of the children, 65 (28.1%), were born with low birth weight in this population. Among women who delivered low-birth-weight children, 53.8% of their husbands were unskilled employees (compared to women who delivered normal-weight children (47.6%), but the differences were not statistically significant (Table 1).

Women delivering low-birth-weight children had higher median values of B6, B12, folate, and homocysteine but lower MMA compared to women who delivered normal-weight children. Women who delivered babies with LBW had a lower sum of skinfold. (Table 2).

We found a significantly weak negative correlation between homocysteine, vitamin B12 (−0.30), and homocysteine and folate (−0.132). There was a weak correlation between MMA and vitamin B12 levels (0.03). (Table 3) Low plasma vitamin B12 concentration, folate deficiency, and B6 deficiency were observed in 48.5%, 42.0%, and 10.4% of the women, respectively. Elevated MMA and elevated homocysteine (>10 µmol/L) were observed among 73.6% and 6.1% of the women, respectively.

Table 4 shows the associations between maternal vitamin B12 and folate status with birth weight. We did not find any significant association between folate and vitamin B12 with birth weight. Composite B12 status measured using Fedosov ‘s formula did not show any significant association with birth weight. The model was adjusted for maternal age, parity, maternal adiposity, gestational diabetes mellitus, and socioeconomic status.

The offspring of the mothers in the lowest quartile of B6 showed a significant association with low birth weight compared to the offspring of the mothers in the highest quartile (OR = 2.80 (1.17, 6.68), *p* = 0.02). No such association was seen between B12 or folate levels with birth weight (Table 5).

Table 6 shows significant associations between maternal vitamin B6 status and preterm delivery, but the association disappears after adjusting for confounders. We did not find any significant association between folate and Vitamin B12 with preterm delivery. Composite B12 status measured using Fedosov’s formula also showed no significant association. The model was adjusted for maternal age, parity, maternal adiposity, gestational diabetes mellitus, BMI, socioeconomic status, and sex of the child.

We found that those with impaired folate status (high homocysteine and low folic acid levels) were twice at risk of low birth weight (OR 1.95 (95% CI 1.29, 3.07) *p* = 0.002) (Supplemental Table S1).

## 4. Discussion

In this study, we investigated the effects of vitamin B6, B12, and folate deficiency on neonatal birth weight. Our findings reveal a significant association between vitamin B6 levels and impaired folate levels with low birth weight.

In our study, B12 status was not associated with birth weight. There have been several other studies that have not found any association between B12 and birth weight [19]. Considering the coenzymatic role of B12, it may not be as influential as nutrients such as folate that serve as a methyl donor in one-carbon metabolism. A randomized controlled trial in Bangalore randomized mothers to receive oral 50 mcg B12 or placebo from 14 weeks of pregnancy; they did not find any difference in birth weight [20]. In contrast, several studies have reported an association between B12 levels and low birth weight [20,21]. A recent systematic review in India showed evidence for an association between B12 deficiency and low birth weight, small for gestational age, and intrauterine growth retardation (IUGR). The review evaluated three prospective cohort studies, one case–control study, and one related to dietary intake of B12 [22]. A systematic review by Rogne et al. found no linear association between B12 and birth weight, but those with B12 less than <148 pmol/L were associated with low birth weight [23].

In our study, methylmalonic acid, a functional marker for B12 deficiency, was not associated with birth weight. In vitamin B12 deficiency, increased concentrations of methyl malonyl-CoA are hydrolyzed and lead to increased amounts of MMA. However, our study did not find a negative correlation between vitamin B12 and MMA. This has been the case in some recent studies where patients with a decreased serum B12 concentration still had normal MMA concentrations [24]. There have been studies where MMA has not been associated with the child’s birth weight [25]. The findings from these studies, although statistically significant, may not be clinically significant due to the small effect sizes. However, given the long-term risks of LBW, further cohort studies with a greater sample size are required to establish this association. Randomized controlled trials supplementing vitamin B12 from preconception to delivery are required to understand the role of vitamin B12 in reducing low birth weight.

Our analysis showed a significant negative correlation between folate and homocysteine. Homocysteine levels were negatively associated with the child’s birth weight (−0.02, *p* = 0.09). We found a significant association between low birth weight and impaired folate status (low folate and raised homocysteine). Two well-established cohorts in South India, the Pune Maternal Nutrition Study (PMNS) and Parthenon Cohort Study, found that offspring birth weight was inversely related to maternal homocysteine concentration [26]. At the same time, there was a strong association between folate levels and foods rich in micronutrients [7]. In Australia, women who developed IUGR had reduced red blood cell (RBC) folate and increased plasma homocysteine concentrations compared with controls [27]. The evidence has been inconsistent. Hogeveen et al. report that birth weight was related negatively to maternal homocysteine (r = –0.12) but not related to maternal B12, methylmalonic acid, and folate [28]. A study in rural India reported no relationship between maternal plasma vitamin B12 concentration and offspring size but showed an association with folate levels [29].

Another important finding of our study is that low vitamin B6 levels were significantly associated with low birth weight; however, there was no negative correlation between B6 and homocysteine. Studies investigating the effect of B6 on LBW are scarce. An epigenetics study showed that higher maternal B6 concentrations were associated with offspring DNA methylation levels at the MEG3 DMR (*p* < 0.01) [30]. Supplementation with folic acid and B6 in women with hyperhomocysteinemia and a history of preeclampsia or fetal growth restriction showed that birth weights had increased; 2867 ± 648 g compared with 1088 ± 570 g in the previous pregnancies [31]. In a meta-analysis of three studies, vitamin B6 supplementation demonstrated a significant positive effect on birth weight. (difference = 217 g [95% CI 130, 304]); however, these studies had a very small sample size [32].

The majority of our participants had poor vitamin B12 status and folate levels. Low B12 levels in mothers perpetuate low B12 levels in infants, which are further triggered by a diet low in B12 levels. The National Nutritional Anemia Prophylaxis Program advocates folate treatment for pregnant women, and yet we found low folate levels in our participants. Vitamin B12 deficiency and folate deficiency increase homocysteine levels and are associated with adverse fetal development. This could be attributed to one-carbon metabolism (OCM), which is essential for cell proliferation, differentiation, and development, and folate, vitamins B12 and B6 play a key role in OCM. Further studies using standardized cut-offs and large numbers of participants are needed to elucidate the impact of B6, folate, vitamin B12, MMA, and homocysteine on neonatal outcomes.

Thus, individual micronutrient deficiency may not hold the key to problems related to low birth weight. The answer would be an integration of solutions. Katre et al. examined the effect of multiple nutrient deficiencies on parameters of one-carbon metabolism in Indian women and compared these with US women. Indian women had lower plasma levels of B12, folate, and B6 and lower plasma levels of essential amino acids and glutathione. The concentration of amino acids, serine, glycine, and homocysteine in the plasma was higher in the Indian subjects. They concluded that the observed changes in amino acids and markers of one-carbon metabolism were likely the result of marginal protein intake and lower B12, folate, and B6 status [33]. Hence, it is important to look at integrated responses during pregnancy, as such studies will allow the evaluation of the relationship between perturbations in one-carbon metabolism and maternal and fetal health.

The strengths of our study include its prospective study design and the use of a sensitive and specific diagnostic method. In addition, we used both direct and functional biomarkers of B12 and folate. We assessed the concentration of appropriate functional markers, such as homocysteine and methylmalonic acid for vitamin B12, which provide greater sensitivity than standalone assays. Some of the limitations of the study are that we did not analyze some important contributors such as dietary intake or dosage and duration of folic acid supplements that the pregnant women may have consumed during pregnancy; these factors might have a confounding effect on the nutrient status of mother and the birth outcomes as well.

There is a growing need to counsel pregnant women on the right diets to ensure adequate vitamins B12 and B6 and folate are consumed. Vitamin B6 levels and impaired folate status were associated with low birth weight in our study. It is essential to conduct large-scale studies investigating the combined role of nutrients involved in OCM to guide the development of intervention. Such steps can ensure intake of folate and micronutrients during preconception and pregnancy to prevent low birth weight.

## Figures and Tables

**Table 1:** Baseline characteristics of pregnant women with and without LBW children.

Characteristics of the Cohort	Categories	Low Birth Weight		Total	*p*-Value
		Yes (*n* = 65)	No (*n* = 166)	Mean or *n* (%)	
Age (years)	Mean ± SD	24.6 ± 3.8	24.1 ± 4.3	24.2 ± 4.2	0.351
Gestational Age at the Time of Sample Collection (weeks)	Mean ± SD	27.8 ± 2.3	27.5 ± 2.2	27.6 ± 2.2	0.354
Religion, *n* (%)	Hinduism	31 (47.6)	68 (40.9)	99 (42.8)	0.567
	Christianity	3 (4.61)	6 (3.61)	9 (3.8)	0.314
	Islam	31 (47.6)	92 (55.4)	123 (53.2)	0.592
Participant Education, *n* (%)	Illiterate/Primary/Middle School	16 (24.6)	41 (24.6)	57 (24.6)	0.735
	High School	25 (38.4)	72 (43.3)	97 (41.9)	0.698
	Pre-University College or Graduation	24 (36.9)	53 (31.9)	77 (33.3)	0.432
Husband’s Education, *n* (%)	Illiterate/Primary/Middle School	25 (38.5)	69 (41.6)	96 (41.8)	0.969
	High School	27 (41.5)	64 (38.6)	91 (39.4)	0.999
	Pre-University College or Graduation	13 (20)	31 (18.7)	44 (19.0)	0.999
Participant’s Occupation, *n* (%)	Unemployed	62 (95.4)	152 (91.6)	214 (92.6)	
	Employed	3 (4.6)	14 (8.4)	17 (7.4)	0.325
Husband’s Occupation, *n* (%)	Unskilled	35 (53.8)	79 (47.6)	114 (49.4)	0.629
	Semi-skilled	18 (27.7)	48 (28.9)	66 (28.6)	0.347
	Skilled/ Professional	12 (18.5)	39 (23.5)	51 (22.1)	0.646
Socioeconomic Class, *n* (%)	Lower	43 (66.2)	107 (64.5)	150 (64.9)	0.808
	Middle	22 (33.8)	59 (35.5)	81 (35.1)	
Gravida, *n* (%)	One	27 (41.5)	57 (34.3)	84 (36.4)	0.565
	Two	24 (36.9)	72 (43.4)	96 (41.6)	0.290
	Three or more	14 (21.5)	37 (22.3)	51 (22.1)	0.566
Parity, *n* (%)	Nulliparous	29 (44.6)	66 (39.8)	95 (41.1)	0.574
	Primiparous	32 (49.2)	83 (50)	115 (49.8)	0.668
	Multiparous	4 (6.2)	17 (10.2)	21 (9.1)	0.297
Sex of Child, *n* (%)	Male	26 (40)	88 (53)	114 (49.4)	0.077
	Female	39 (60)	78 (47)	117 (50.6)	

**Table 2:** Biochemical and nutritional markers of pregnant women with and without LBW children.

Characteristics of the Cohort	Categories	Low Birth Weight		Total	*p*-Value
		Yes (*n* = 65)	No (*n* = 166)		
Anemia, *n* (%)	Present	27 (41.5)	73 (44)	100 (43.3)	0.737
	Absent	38 (58.5)	93 (56)	131 (56.7)	
Gestational Diabetes, *n* (%)	Present	10 (15.4)	34 (20.5)	44 (19)	0.377
	Absent	55 (84.6)	132 (79.5)	187 (81)	
Vitamin B12 (pmol/L)	Median (IQR)	232.2 (184.8)	221.4 (106.9)	222.6 (134.7)	
Homocysteine (μmol/L)	Median (IQR)	6.8 (2.3)	6.5 (2.3)	6.6 (2.3)	
MMA (μmol/L)	Median (IQR)	0.34 (0.3)	0.38 (0.1)	0.38 (0.23)	
Serum Folate (nmol/L)	Median (IQR)	6.7 (7.8)	4.7 (7.7)	5.1 (7.5)	
Vitamin B6 (nmol/L)	Median (IQR)	71 (47.7)	61 (45.8)	64.7 (47.2)	
Had Received Iron and Folic Acid Supplementation at the Time of Interview (IFA)	Yes	41 (63.1)	121 (72.9)	162 (70.1)	0.144
	No	24 (36.9)	45 (27.1)	69 (29.9)	
Sum of Skinfold Thickness (mm)	Mean ± SD	43.66 ± 13.7	48.44 ± 13.4	47.10 ± 13.6	0.018
Body Mass Index (BMI)	Normal (18.5–22.9)	25 (38.5)	53 (31.9)	78 (33.8)	0.221
	Underweight (<18.5)	7 (10.8)	8 (4.8)	15 (6.5)	0.089
	Overweight (23–24.9)	8 (12.3)	30 (18.1)	38 (16.5)	0.300
	Obese (≥25)	25 (38.5)	75 (45.2)	100 (43.3)	0.628

**Table 3:** Correlation between vitamin B12, MMA, homocysteine, and folate.

Variable	Pearson’s Correlation	*p*-Value
B12 (pmol/L) and MMA(μmol/L)	0.03	0.63
B12 (pmol/L) and Hcys(μmol/L)	−0.300	>0.001
Folate (nmol/L) and Hcys(μmol/L)	−0.132	0.04
B6 (pmol/L) and Hcys(μmol/L)	0.05	0.42

**Table 4:** Association between maternal B12 and folate markers with neonatal birth weight in the MAASTHI cohort.

	Unadjusted		Adjusted	
Exposure	β(SE)	*p*-Value	β(SE)	*p*-Value
Vitamin B12	4.83 (0.0)	0.813	0.00 (0.0)	0.473
MMA	0.15 (0.16)	0.346	0.18 (0.16)	0.265
Homocysteine	−0.02 (0.01)	0.091	−0.02 (0.01)	0.118
Folate	−0.004 (0.05)	0.388	−0.003 (0.05)	0.562
Composite B12 (cB12)	0.08 (0.107)	0.415	0.12 (0.10)	0.252

Adjusted for maternal age, parity, maternal adiposity, gestational diabetes mellitus, and socioeconomic status.

**Table 5:** Odds ratios of low birth weight for vitamins B6, B12 and folate concentrations.

	OR (95% CI)	*p*-Value	OR (95% CI)	*p*-Value	OR (95% CI)	*p*-Value	Reference
Vitamin B6 (nmol/L) *	Quartile 1		Quartile 2		Quartile 3		Quartile 4
Unadjusted	2.33 (1.01, 5.35)	0.004	1.59 (0.71, 3.54)	0.25	1.40 (0.64, 3.07)	0.39	1
Model 1 †	2.66 (1.12, 6.29)	0.02	1.94 (0.83, 4.52)	0.12	1.69 (0.73, 3.93)	0.21	1
Model 2 ‡	2.80 (1.17, 6.68)	0.02	1.97 (0.84, 4.60)	0.11	1.75 (0.74, 4.11)	0,19	1
Vitamin B12 (pmol/L) *	Quartile 1		Quartile 2		Quartile 3		Quartile 4
Unadjusted	1.26 (0.38, 4.15)	0.70	0.81 (0.34, 1.90)	0.62	0.92 (0.39, 2.18)	0.86	1
Model 1 †	1.51 (0.44, 5.10)	0.50	0.98 (0.40, 2.39)	0.97	0.90 (0.37, 2.17)	0.81	1
Model 2 ‡	2.09 (0.59, 7.40)	0.25	1.41 (0.55, 3.60)	0.47	1.14 (0.45, 2.86)	0.77	1
Folate (nmol/L) *	Quartile 1		Quartile 2		Quartile 3		Quartile 4
Unadjusted	1.62 (0.69, 3.81)	0.26	1.45 (0.64, 3.31)	0.37	0.61 (0.28, 1.35)	0.23	1
Model 1 †	1.54 (0.63, 3.70)	0.33	1.41 (0.60, 3.27)	0.42	0.61 (0.27, 1.37)	0.23	1
Model 2 ‡	1.99 (0.77, 5.12)	0.15	1.70 (0.71, 4.04)	0.22	0.72 (0.31, 1.68)	0.45	1

* Unadjusted. † Adjusted for maternal age, parity, maternal adiposity, gestational diabetes mellitus, socioeconomic status, and sex of child. ‡ Adjusted for maternal age, parity, maternal adiposity, gestational diabetes mellitus, socioeconomic status, and two other vitamins.

**Table 6:** Association between maternal B6, B12, and folate with preterm delivery at birth in the MAASTHI cohort.

	Unadjusted		Adjusted	
Exposure	OR (95% CI)	*p*-Value	OR (95% CI)	*p*-Value
Vitamin B12	0.99 (0.9, 1.00)	0.43	0.99 (0.99, 1.00)	0.414
MMA	7.09 (0.42, 1.18)	0.17	6.89 (0.41, 1.14)	0.178
Homocysteine	1.95 (0.72, 1.16)	0.49	0.91 (0.70, 1.18)	0.490
Folate	1.03 (0.96, 1.11)	0.29	1.03 (0.95, 1.10)	0.409
Composite B12	0.79 (0.15, 4.21)	0.79	0.78 (0.13, 4.53)	0.786
Vitamin B6	1.00 (1.0, 1.01)	0.05	1.0 (0.9, 1.01)	0.462

Adjusted for maternal age, parity, maternal adiposity, gestational diabetes mellitus, BMI, socioeconomic status, and sex of the child.

## Data Availability

The datasets used and analyzed during the current study are available from the corresponding author upon reasonable request.

## Supplementary Materials

The following supporting information can be downloaded at: https://www.mdpi.com/article/10.3390/nu15071793/s1, Table S1: Associations between the impaired folate status with the prevalence of low birth weight.
