# Supplementary material for: Vitamin B6 Levels and Impaired Folate Status but Not Vitamin B12 Associated with Low Birth Weight: Results from the MAASTHI Birth Cohort in South India
Source: Nutrients. 2023 Apr 6;15(7):1793. doi: 10.3390/nu15071793 (PMC10096757; doi:10.3390/nu15071793)
Supplement: Supplementary file 1 [file nutrients-15-01793-s001.zip › nutrients-2255878-supplementary.pdf]

Supplementary Table S1. Associations between the Impaired folate status with the prevalence of low birth weight

	Causal Model with IPTW		
	OR	95% CI	p
**Impaired folate status**	1.95	1.29, 3.07	**0.002**

Adjusted for maternal age, parity, maternal adiposity, gestational diabetes mellitus, BMI, socioeconomic status, and sex of the child
